# Auto-antibodies against type I IFNs in > 10% of critically ill COVID-19 patients: a prospective multicentre study

**DOI:** 10.1186/s13613-022-01095-5

**Published:** 2022-12-31

**Authors:** Romain Arrestier, Paul Bastard, Thibaut Belmondo, Guillaume Voiriot, Tomas Urbina, Charles-Edouard Luyt, Adrian Gervais, Lucy Bizien, Lauriane Segaux, Mariem Ben Ahmed, Raphaël Bellaïche, Taï Pham, Zakaria Ait-Hamou, Damien Roux, Raphael Clere-Jehl, Elie Azoulay, Stéphane Gaudry, Julien Mayaux, Nicolas Fage, Hafid Ait-Oufella, Elsa Moncomble, Mélodie Parfait, Karim Dorgham, Guy Gorochov, Armand Mekontso-Dessap, Florence Canoui-Poitrine, Jean-Laurent Casanova, Sophie Hue, Nicolas de Prost

**Affiliations:** 1 Service de Médecine Intensive Réanimation, Service de Réanimation Médicale, Hôpital Henri Mondor, Hôpitaux Universitaires Henri Mondor, Assistance Publique-Hôpitaux de Paris, Créteil, 94010 Paris, Cedex, France; 2 Groupe de Recherche Clinique CARMAS, Faculté de Santé de Créteil, Université Paris Est Créteil, Créteil, 94010 Paris, Cedex, France; 3 INSERM, IMRB, Université Paris Est Créteil, Créteil, 94010 Paris, Cedex, France; 4 Laboratory of Human Genetics of Infectious Diseases, Necker Branch, INSERM U1163, Necker Hospital for Sick Children, Paris, France; 5 Imagine Institute, University of Paris, Paris, France; 6 St. Giles Laboratory of Human Genetics of Infectious Diseases, Rockefeller Branch, The Rockefeller University, New York, NY USA; 7 Département d’Hématologie et d’Immunologie Biologiques, Assistance Publique-Hôpitaux de Paris (AP-HP), Groupe Hospitalo-Universitaire Chenevier Mondor, Créteil, 94010 Paris, France; 8 Service de Médecine Intensive-Réanimation, Hôpital Tenon, Assistance Publique-Hôpitaux de Paris (AP-HP), Paris, France; 9 Service de Médecine Intensive-Réanimation, Hôpital Saint-Antoine, Assistance Publique-Hôpitaux de Paris (AP-HP), Paris, France; 10 Service de Médecine Intensive Réanimation, Sorbonne Université, Hôpitaux Universitaires Pitié Salpêtrière-Charles Foix, Assistance Publique-Hôpitaux de Paris (AP-HP), Paris, France; 11 INSERM UMRS_1166-iCAN, Institute of Cardiometabolism and Nutrition, Paris, France; 12 Unité de Recherche Clinique AP-HP, Hôpitaux Henri-Mondor, 94010 Creteil, Cedex, France; 13 Service d’Anesthésie-Réanimation Chirurgicale, Assistance Publique-Hôpitaux de Paris, Hôpitaux Universitaires Henri Mondor, 94000 Créteil, France; 14 Service de Médecine Intensive-Réanimation, AP-HP, Hôpital de Bicêtre, DMU 4 CORREVE Maladies du Cœur et des Vaisseaux, FHU Sepsis, Groupe de Recherche Clinique CARMAS, Le Kremlin-Bicêtre, France; 15 Service de Médecine Intensive-Réanimation, Hôpital Cochin, Assistance Publique-Hôpitaux de Paris (AP-HP), Centre & Université de Paris, Paris, France; 16 Médecine Intensive Réanimation, AP-HP, Hôpital Louis Mourier, DMU ESPRIT, 92700 Colombes, France; 17 Service de médecine intensive et réanimation, Hôpital Saint-Louis, Assistance Publique Des Hôpitaux de Paris, Paris, France; 18 Département de réanimation médico-chirurgicale, APHP Hôpital Avicenne, Bobigny, France; 19 Groupe Hospitalier Pitié Salpêtrière, Assistance Publique Hôpitaux de Paris, Service de Pneumologie et Réanimation Médicale, Paris, France; 20 Sorbonne Université, Inserm, Centre d’Immunologie et des Maladies Infectieuses (CIMI-Paris), 75013 Paris, France; 21 Département d’Immunologie, Assistance Publique Hôpitaux de Paris (AP-HP), Hôpital Pitié-Salpêtrière, 75013 Paris, France

**Keywords:** COVID-19, Interferon, Auto-antibodies, Acute respiratory distress syndrome

## Abstract

**Background:**

Auto-antibodies (auto-Abs) neutralizing type I interferons (IFN) have been found in about 15% of critical cases COVID-19 pneumonia and less than 1% of mild or asymptomatic cases. Determining whether auto-Abs influence presentation and outcome of critically ill COVID-19 patients could lead to specific therapeutic interventions. Our objectives were to compare the severity at admission and the mortality of patients hospitalized for critical COVID-19 in ICU with *versus* without auto-Abs.

**Results:**

We conducted a prospective multicentre cohort study including patients admitted in 11 intensive care units (ICUs) from Great Paris area hospitals with proven SARS-CoV-2 infection and acute respiratory failure. 925 critically ill COVID-19 patients were included. Auto-Abs neutralizing type I IFN-α2, *β* and/or ω were found in 96 patients (10.3%). Demographics and comorbidities did not differ between patients with *versus* without auto-Abs. At ICU admission, Auto-Abs positive patients required a higher FiO_2_ (100% (70–100) vs. 90% (60–100), *p* = 0.01), but were not different in other characteristics. Mortality at day 28 was not different between patients with and without auto-Abs (18.7 vs. 23.7%, *p* = 0.279). In multivariable analysis, 28-day mortality was associated with age (adjusted odds ratio (aOR) = 1.06 [1.04–1.08], *p* < 0.001), SOFA score (aOR = 1.18 [1.12–1.23], *p* < 0.001) and immunosuppression (aOR = 1.82 [1.1–3.0], *p* = 0.02), but not with the presence of auto-Abs (aOR = 0.69 [0.38–1.26], *p* = 0.23).

**Conclusions:**

In ICU patients, auto-Abs against type I IFNs were found in at least 10% of patients with critical COVID-19 pneumonia. They were not associated with day 28 mortality.

**Supplementary Information:**

The online version contains supplementary material available at 10.1186/s13613-022-01095-5.

## Background

Since the beginning of the pandemic, SARS-CoV-2 infected more than 500 million individuals and has been responsible for at least 6.2 million deaths [1] with recent estimates reaching 18.2 million deaths [2]. SARS-CoV-2 infection leads to a broad spectrum of manifestations with vast inter-individual variability. Some patients are asymptomatic while others develop severe pneumonia potentially requiring intensive care unit (ICU) admission. Demographic factors associated with the severity of coronavirus disease 2019 (COVID-19) have been extensively studied, age being by far the most impactful risk factor, while male gender, diabetes, obesity, hypertension and cardiovascular comorbidities are much more modest risk factors [3]. The protective role of type I interferons (IFNs) immunity during SARS-CoV-2 infection was documented by the observation of life-threatening COVID-19 pneumonia in patients with inborn errors of immunity affecting Toll-like receptor 3 (TLR3) or TLR7-dependent type I IFNs induction and amplification, in 1–5% of cases of critical COVID-19 pneumonia [4–6]. Type I IFNs are potent anti-viral molecules that activate interferon-stimulated genes (ISGs), leading to the anti-viral response [7]. Autoimmune phenocopy of inborn errors of type I IFN-dependent immunity were also shown to underlie life-threatening COVID-19 pneumonia. Circulating IgG auto-antibodies (auto-Abs) neutralizing IFN-α2 and/or IFN-ω (10 ng/mL) were found in 10% of critical COVID-19 cases in an international cohort, as compared with 0% of mildly/asymptomatic cases and 0.3% of uninfected individuals [5]. Auto-Abs neutralizing 100-fold lower concentrations of IFN-α2 and/or IFN-ω (100 pg/mL; in 1:10 dilutions of plasma) were further detected in 13.6% of critically ill patients with COVID-19 and 18% of the deceased, while auto-Abs to IFN-β were found in another 1% of critical patients [8]. Auto-Abs were mostly found in men and in patients over the age of 65 years [5]. Several cohort series [4, 9–20] replicated these findings.

While it is now clearly established that these auto-Abs pre-exist to infection and are causal of critical COVID-19 pneumonia, it remains unclear if they underlie a worse clinical presentation or outcome. Determining whether auto-Abs neutralizing type I INFs are associated with mortality in critically ill patients with COVID-19 has important clinical implications. In addition, their detection could trigger specific therapeutic interventions including plasma exchange therapy [21], monoclonal antibodies or recombinant IFN-β1 [9].

In this study, we conducted a multicenter cohort of COVID-19 patients requiring ICU admission and aimed to: (1) compare the severity of patients at admission and (2) compare the mortality of patients with *versus* without auto-Abs neutralizing type I INFs.

## Methods

### Study design and participants

We conducted an observational prospective multicentre study (ANTICOV; NCT04733105) in 11 ICUs of the Great Paris area hospitals between March 31st 2020 and May 1st 2021. Inclusion criteria were as follows: age ≥ 18 years, SARS-CoV-2 infection confirmed by a positive reverse transcriptase-polymerase chain reaction (RT-PCR), patient admitted in the ICU for acute respiratory failure (SpO2 ≤ 90% and need for supplemental oxygen or any kind of ventilator support). Patients with SARS-CoV-2 infection but no acute respiratory failure were not included in the study. The study was approved by the Comité de Protection des Personnes Nord-Ouest IV (N° EudraCT/ID-RCB: 2020-A03009-30). Informed consent was obtained from all patients or their relatives.

Demographics, clinical and laboratory variables were recorded upon ICU admission and during ICU stay. Patients’ frailty was assessed using the Clinical Frailty Scale [22]. The severity of the disease upon ICU admission was assessed using the World Health Organization (WHO) 10-point progression scale [23], the sequential organ failure assessment (SOFA score) [24], and the Simplified Acute Physiology Score (SAPS) II score [25]. Acute respiratory distress syndrome (ARDS) was defined according to the Berlin definition [26]. The primary clinical endpoint of the study was day-28 mortality. Follow-up ended at day 90 after ICU admission.

### Functional evaluation of anti-cytokine auto-Abs by luciferase reporter assays

Auto-Abs positivity was assessed on serum samples collected during the first week of ICU admission. The blocking activity of anti-IFN-α2 and anti-IFN-ω auto-Abs was determined with a reporter luciferase activity, as previously described [8]. Briefly, HEK293T cells were transfected with a plasmid containing the *Firefly* luciferase gene under the control of the human *ISRE* promoter in the pGL4.45 backbone, and a plasmid constitutively expressing *Renilla* luciferase for normalization (pRL-SV40). Cells were transfected in the presence of the X-tremeGene9 transfection reagent (Sigma-Aldrich, ref. number 6365779001) for 24 h. Cells in Dulbecco’s modified Eagle medium (DMEM, Thermo Fisher Scientific) supplemented with 2% foetal calf serum (FCS) and 10% healthy control or patient serum (after inactivation at 56 °C, for 20 min) were either left unstimulated or were stimulated with IFN-α2 (Miltenyi Biotec, ref. number 130–108-984), IFN-ω (Merck, ref. number SRP3061), at 10 ng/mL or 100 pg/mL, or IFN-β (Miltenyi Biotec, ref. number: 130-107-888) at 10 ng/mL, for 16 h at 37 °C. Each sample was tested once for each cytokine and dose. Finally, cells were lysed for 20 min at room temperature and luciferase levels were measured with the Dual-Luciferase® Reporter 1000 assay system (Promega, ref. number E1980), according to the manufacturer’s protocol. Luminescence intensity was measured with a VICTOR-X Multilabel Plate Reader (PerkinElmer Life Sciences, USA). *Firefly* luciferase activity values were normalized against *Renilla* luciferase activity values. These values were then normalized against the median induction level for non-neutralizing samples, and expressed as a percentage. Samples were considered neutralizing if luciferase induction, normalized against *Renilla* luciferase activity, was below 15% of the median values for controls tested the same day.

### Anti-nuclear antibody assay

Anti-nuclear antibodies (ANA) were screened on serum samples with indirect immunofluorescent assay on conventional HEp-2 substrate in 812/925 patients of the cohort.

### Statistics

Descriptive results are presented as medians (1st–3rd quartiles) for continuous variables and as numbers with percentage for categorical variables. Unadjusted between-group (i.e., according to auto-Abs status and vital status at 90-day) comparisons were performed in the whole cohort and in subgroups of patients (i.e., in patients with positive auto-Abs and in women) using Student’s *t* tests or Mann–Whitney tests for continuous variables, and Chi^2^ or Fisher’s exact tests for categorical variables, as appropriate. Adjusted analyses of the association between auto-Abs and 28-day mortality relied on multivariable logistic regression models, adjusting for age, SOFA score at ICU admission, gender and major comorbidities shown to be associated with mortality [3], computing adjusted odds ratios (aOR) along with their 95% confidence intervals (CI). Calibration of the models was evaluated using the Hosmer–Lemeshow test. Two-tailed *p*-values < 0.05 were considered statistically significant. Analyses were performed using Stata V16.0 statistical software (StataCorp, College Station, TX, USA), and R 3.6.3 (R Foundation for Statistical Computing, Vienna, Austria).

## Results

### Prevalence of auto-Abs against type I IFN and ICU admission characteristics

A total of 925 critically ill COVID-19 patients were included in the cohort between March 2020 and May 2021 and had serum samples analysed for neutralization ability of anti-IFN auto-Abs. The median age of the patients included in the whole cohort was 62 years, 70% of whom were male. Hypertension (51.2%) and obesity (43.0%) were the most frequent comorbidities, as expected in critically ill COVID-19 patients [27].

We found auto-Abs neutralizing type I IFNs in 96 patients of the cohort (10.3%, 95% CI [8.4–12.3]), in the same range of what was previously reported [5, 8]. Demographics and comorbidities did not differ between patients with and without auto-Abs neutralizing type I IFN (Table 1). The proportion of males was not statistically different between positive and negative auto-Abs patients (78.1 vs. 69.6%, *p* = 0.083) and the proportion of positive auto-Abs patients was not different across gender (11.5% (*n* = 75/652) vs. 7.7% (*n* = 21/273)), contrasting with studies initially reporting that anti-IFN auto-Abs were found almost uniquely (94%) in men [5], while more recent studies showed a less obvious trend [8].

**Table 1:** Demographics and characteristics of patients with severe SARS-CoV-2 infection (*n* = 925) at intensive care unit admission, according to the presence of auto-antibodies against type I IFNs

	Total	Negative anti-IFN auto-Abs	Positive anti-IFN auto-Abs	*p*-value^a^
	*N* = 925	*N* = 829	*N* = 96	
Comorbidities				
Age^b^	62 (53;69.7)	62.0 (53;69.5)	63.8 (53.9;70.5)	0.668
Gender				0.083
Male^b^	652 (70.5)	577 (69.6)	75 (78.1)	
Female	273 (29.5)	252 (30.4)	21 (21.9)	
Obesity^b^ (*N* = 861)	370 (43.0)	335 (43.4)	35 (39.3)	0.463
Diabetes^b^	301 (32.5)	278 (33.5)	23 (24.0)	0.058
Congestive heart failure^c^	94 (10.2)	85 (10.3)	9 (9.4)	0.787
Vasculopathy	101 (10.9)	87 (10.5)	14 (14.6)	0.224
Hypertension^b^	474 (51.2)	429 (51.8)	45 (46.9)	0.366
COPD	62 (6.7)	60 (7.2)	2 (2.1)	0.053
Chronic kidney disease^d^	106 (11.5)	97 (11.7)	9 (9.4)	0.498
ESRD requiring dialysis	42 (4.5)	37 (4.5)	5 (5.2)	0.794
Cirrhosis	13 (1.4)	11 (1.3)	2 (2.1)	0.636
Current smoking	124 (13.4)	107 (12.9)	17 (17.7)	0.191
Immunosuppression^b^	100 (10.8)	95 (11.5)	5 (5.2)	0.080
Solid cancer	31 (3.4)	28 (3.4)	3 (3.1)	1.000
HIV infection	15 (1.6)	15 (1.8)	0 (0)	0.388
Haematological malignancy	17 (1.8)	17 (2.1)	0 (0)	0.243
Clinical Frailty Scale	3 (2;4)	3 (2;4)	3 (2;3)	0.173
Characteristics at ICU admission				
SAPS II (*N* = 914)	34 (26;44)	34 (26;44)	34.5 (29;44)	0.433
SOFA score^b^ (*N* = 917)	4 (2;6)	4 (2;6)	4 (2;7)	0.343
WHO CPS(*N* = 920)	6 (6;8)	6 (6;8)	7 (6;8)	0.188
Time from first symptoms to ICU admission (days)	9 [6–12]	9 [6–12]	8 [6–12]	0.531
Admission period				**0.004**
First wave^e^	140 (15.1)	115 (13.9)	25 (26)	
Second wave^f^	785 (84.9)	714 (86.1)	71 (73.9)	
Temperature (*N* = 857)	37 (36.9;38)	37 (36.9;38)	37.4 (36.7;38.1)	0.299
FiO_2_ (%) (*N* = 908)	90 (60;100)	90 (60;100)	100 (70;100)	**0.010**
PaO_2_ (mmHg) (*N* = 906)	73 (61;91)	72 (61;91)	76 (63.5;89)	0.259
PaO_2_/FiO_2_ ratio (*N* = 903)	0.96 (0.7;1.4)	0.97 (0.70;1.4)	0.9 (0.7;1.3)	0.427
PaCO_2_ (mmHg) (*N* = 895)	37 (32;43)	37 (32;43)	38 (34;45)	0.118
Arterial lactate (mmol/L) (*N* = 889)	1.4 (1.2;1.9)	1.4 (1.2;1.9)	1.5 (1.1;2.1)	0.898
White blood cell counts (G/L) (*N* = 920)	8.80 (6.1;12.8)	8.5 (5.9;12.3)	11.41 (8.3;14.7)	** < 0.0001**
Neutrophil counts (G/L) (*N* = 736)	7.8 (5.2;11.3)	7.44 (5;11)	10.2 (6.4;13.8)	**0.0002**
Lymphocyte counts (G/L) (*N*=733)	0.7 (0.5;1)	0.7 (0.5;1)	0.7 (0.5;1.1)	0.976
Monocyte counts (G/L) (*N* = 732)	0.4 (0.2;0.6)	0.40(0.2;0.6)	0.5 (0.3;0.8)	**0.008**
Plasma creatinine level (µmol/L) (*N* = 922)	76 (60;108)	76 (59;109)	81 (64;103.5)	0.297
D-dimers (ng/mL) (*N* = 510)	1344.5 (846;2614)	1343 (837;2511)	1380 (937;3488)	0.258
Corticosteroid	44 (4.8)	42 (5.1)	2 (2.1)	0.307
Antibiotic treatment^b^	635 (68.6)	564 (68.0)	71 (74.0)	0.236
Oxygen therapy	51 (5.5)	46 (5.6)	5 (5.2)	0.890
High flow oxygen therapy	525 (56.8)	474 (57.2)	51 (53.1)	0.448
Invasive mechanical ventilation	441 (47.7)	388 (46.8)	53 (55.2)	0.119
ARDS	862 (93.2)	772 (93.1)	90 (93.8)	0.818
Shock	178 (19.2)	156 (18.8)	22 (22.9)	0.335
Renal replacement therapy	98 (10.6)	88 (10.6)	10 (10.4)	0.952
Neuromuscular blockade	404 (43.7)	359 (43.4)	45 (46.9)	0.511

Continuous values are shown as median (quartile 1-quartile 3); qualitative values are shown as number (percentage); ^a^*p* values come from Chi^2^, Fisher, Student’s or Mann–Whitney tests, as appropriate; ^b^variable included in the multivariable logistic regression analysis assessing the relationship between auto-antibodies against type I interferon and 28-day mortality (Table 3); ^c^defined as stages III–IV the New York Heart Association classification; ^d^glomerular filtration rate < 60 mL/min/1.73m^2^; ^e^from March 2020 to June 2020; ^f^from July 2020 to May 2021: bolded values are significant at the < 0.05 level

*COPD* chronic obstructive pulmonary disease, *ESRD* end-stage renal disease, NSAI: non-steroidal anti-inflammatory, *SAPS II* Simplified Acute Physiology Score II, *SOFA* Sequential Organ Failure Assessment, *WHO CPS* World Health Organization clinical progression scale, *ARDS* acute respiratory distress syndrome, *RRT* renal replacement therapy, *iNO* inhaled nitric oxide, *ECMO* extra-corporeal membrane oxygenation

At ICU admission, 47.7% of patients (*n* = 441) required invasive mechanical ventilation support and 19.2% of them (*n* = 178) needed vasopressors, with no significant difference between patients with and without auto-Abs neutralizing type I IFN (Table 1). There was also no significant differences between these two groups regarding severity of illness, as assessed using the SAPS II and SOFA scores and the WHO clinical progression scale. Yet, patients with positive auto-Abs neutralizing type I IFN required a higher FiO_2_ at admission than others (100% (70–100) vs. 90% (60–100), *p* = 0.010), but there was no significant difference regarding gas exchange or ventilatory support. Patients with auto-Abs neutralizing type I IFN also had significantly higher granulocytes counts than others, with higher neutrophils and monocytes counts but with similar lymphocytes counts.

### ICU management and outcomes

There were no significant differences regarding patient management and organ support according to the presence of auto-Abs neutralizing type I IFNs (Table 2). Mortality at day 28 (23.2%) was not significantly different in patients with auto-Abs compared to patients without (18.7 vs. 23.7%, *p* = 0.279). Consistently, there was also no significant difference at day 90 (28.1 vs. 30.5%, *p* = 0.630).

**Table 2:** Intensive care management and outcomes of patients with severe SARS-CoV-2 infection (*n* = 925) at intensive care unit admission, according to the presence of auto-antibodies against type I IFNs

	Total	Negative anti-IFN auto-Abs	Positive anti-IFN auto-Abs	*p*-value^a^
	*N* = 925	*N* = 829	*N* = 96	
Duration of hospital stay, days (*N* = 642)	19.5 (11;37)	19.0 (11;37)	22 (13;44)	0.155
Duration of stay in the ICU, days (*N* = 655)	12.0 (6;26)	11.0 (6;25)	13.0 (7;31)	0.157
Invasive mechanical ventilation	644 (69.6)	576 (69.5)	68 (70.8)	0.785
Duration of mechanical ventilation support. days (N = 643)	17 (9;28)	16 (9;28)	18 (9;30.5)	0.557
Ventilator-free days, 28 days (*N* = 583)	1 (0;5)	1 (0;5)	2 (0;6)	0.772
Ventilator-free days, 90 days (*N* = 640)	0.5 (0;4)	0 (0;4)	1 (0;5)	0.578
Ventilator-associated pneumonia (*N* = 643)	457 (71)	406 (70.6)	51 (75)	0.450
Continuous sedation	629 (68)	563 (67.9)	66 (68.8)	0.868
Neuromuscular blockade	620 (67)	553 (66.7)	67 (69.8)	0.543
Prone positioning	632 (68.3)	564 (68)	68 (70.8)	0.577
Inhaled nitric oxide	91 (9.8)	82 (9.9)	9 (9.4)	0.872
ECMO	166 (17.95)	143 (17.3)	23 (23.96)	0.105
Duration of ECMO support, days	17 (7;33)	17 (7;35)	16 (7;28)	0.546
Renal replacement therapy	265 (28.7)	238 (28.7)	27 (28.1)	0.773
Shock	507 (54.8)	455 (54.9)	52 (54.2)	0.914
Duration of vasopressor support, days (*N* = 486)	7 (3;15)	7 (3;15)	8 (3;13)	0.968
Vasopressor-free days, 28 days (*N* = 264)	14 (6;28)	14 (6;28)	16 (8;28)	0.630
Vasopressor-free days, 90 days (*N* = 261)	14 (6;28)	13.5 (6;28)	16 (8;28)	0.541
Dexamethasone	678 (73.3)	614 (74.1)	64 (66.7)	0.121
Hydrocortisone	138 (14.9)	121 (14.6)	17 (17.7)	0.418
Other corticosteroids				0.541
Fludrocortisone	12 (1.3)	10 (1.2)	2 (2.1)	
Methylprednisolone	107 (11.6)	99 (12.0)	8 (8.3)	
Prednisone	17 (1.8)	15 (1.8)	2 (2.1)	
Mortality, 28-day	214 (23.2)	196 (23.7)	18 (18.8)	0.279
Mortality, 90-day	279 (30.3)	252 (30.5)	27 (28.1)	0.630

Continuous values are shown as median (quartile 1-quartile 3); qualitative values are shown as number (percentage); ^a^*P* values come from Fisher’s or Chi^2^ tests, and Student’s *t* tests or Mann–Whitney tests, as appropriate

In multivariable analysis, the variables that were significantly associated with 28-day mortality were age (aOR = 1.06 [1.04–1.08] per year, *p* < 0.001), SOFA score (aOR = 1.18 [1.12–1.23] per point, *p* < 0.001), and immunosuppression (aOR = 1.82 [1.10–3.00], *p* = 0.020). The presence of auto-Abs neutralizing type I IFN was not associated with an increased mortality at 28-day within patients hospitalized in the ICU (aOR = 0.69 [0.38–1.26], *p* = 0.23) (Table 3).

**Table 3:** Multivariable logistic regression analysis of factors associated with mortality at 28 days in patients with severe SARS-CoV-2 infection (*n* = 852)

	Day-28 mortality	
	aOR [95% CI]	*P* value
Anti-IFN auto-antibody	0.69 [0.38–1.25]	0.222
Age, *year*	1.06 [1.04–1.08]	** < 0.0001**
Male gender	1.37 [0.92–2.03]	0.119
Immunosuppression^a^	1.81 [1.10–2.98]	**0.020**
Hypertension	1.11 [0.76–1.61]	0.591
Obesity	1.00 [0.69–1.45]	0.993
Diabetes	0.84 [0.57–1.22]	0.351
SOFA score, per point	1.17 [1.12–1.23]	** < 0.0001**
Antibiotic at ICU admission	1.09 [0.75–1.60]	0.641

*aOR [95% CI]* adjusted odds ratio [95% confidence interval]

Hosmer–Lemeshow Chi^2^ *p*-value: 0.403

^a^Refers to pre-existing immunosuppression; bolded values are significant at the < 0.05 level

### Description of auto-Abs against type I IFN

**Fig. 1:**
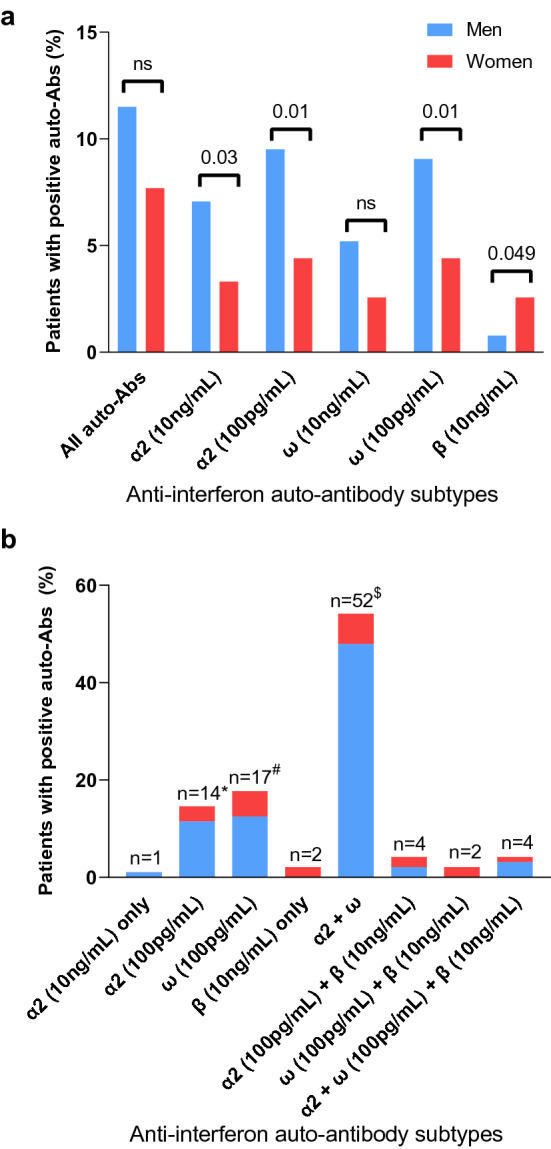
Distribution of auto-antibodies (auto-Abs) to specific sets of type I interferons (IFNs) according to gender. **a** Proportion of specific sets of auto-Abs (several patients have more than one auto-Abs resulting in total number being greater than 96); **b** proportion of combinations of auto-Abs to specific sets of type I IFNs (total numbers add-up to 96); *α*2: auto-Abs anti-IFN-α2; *ω*: auto-Abs anti-IFN-ω; *β*: auto-Abs anti-IFN-β; *including 6 patients who also had anti-IFN-α2 (10 ng/mL) auto-Abs; ^#^including 2 patients who also had anti-IFN-ω (10 ng/mL) auto-Abs; ^$^including 6 patients who had anti-IFN-α2 (100 pg/mL) and anti-IFN-ω (100 pg/mL), 4 patients with anti-IFN-α2 (100 pg/mL + 10 ng/mL) and anti-IFN-ω (10 ng/mL), 35 patients with anti-IFN-α2 (100 pg/mL + 10 ng/mL) and anti-IFN-ω (100 pg/mL + 10 ng/mL), 6 patients with IFN-α2 (100 pg/mL + 10 ng/mL) and anti-IFN-ω (100 pg/mL) and 1 patient with IFN-α2 (10 ng/mL) + anti-IFN-ω (100 pg/mL + 10 pg/mL) auto-Abs; males are shown in blue and females in red; NS: *p*-value > 0.05

**Fig. 2:**
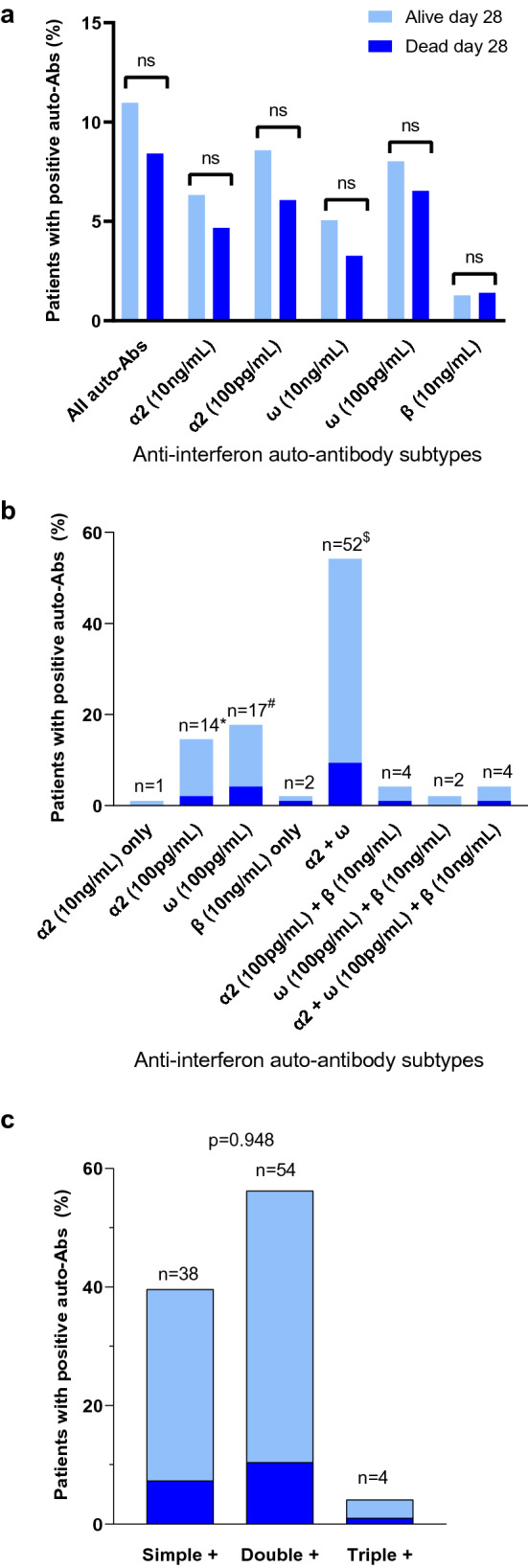
Distribution of auto-antibodies (auto-Abs) to specific sets of type I interferons (IFNs) according to 28-day mortality. **a** Proportion of specific sets of auto-Abs (several patients have more than one auto-Abs resulting in total number being greater than 96; **b** proportion of combinations of auto-Abs to specific sets of type I IFNs (total numbers add-up to 96); *α*2: auto-Abs anti-IFN-α2; *ω*: auto-Abs anti-IFN-ω; *β*: auto-Abs anti-IFN-β; *including 6 patients who also had anti-IFN-α2 (10 ng/mL) auto-Abs; ^#^including 2 patients who also had anti-IFN-ω (10 ng/mL) auto-Abs; ^$^including 6 patients who had anti-IFN-α2 (100 pg/mL) and anti-IFN-ω (100 pg/mL), 4 patients with anti-IFN-α2 (100 pg/mL + 10 ng/mL) and anti-IFN-ω (10 ng/mL), 35 patients with anti-IFN-α2 (100 pg/mL + 10 ng/mL) and anti-IFN-ω (100 pg/mL + 10 ng/mL), 6 patients with IFN-α2 (100 pg/mL + 10 ng/mL) and anti-IFN-ω (100 pg/mL) and 1 patient with IFN-α2 (10 ng/mL) + anti-IFN-ω (100 pg/mL + 10 pg/mL) auto-Abs; **c** distribution of patients with simple, double or triple positive auto-Abs; P value comes from the Fisher exact test. Patients who were alive at 28-day are shown in light blue and patients who were dead at 28-day are shown in dark blue (**b**, **c**); NS: *p*-value > 0.05

The distribution of auto-Abs to the different subtypes of type I IFNs is shown in Figs. 1 and 2. As previously described [8], the capacity of auto-Abs to neutralize type I IFNs was tested at two in vitro concentrations of IFN for auto-Abs against IFN-α2 and IFN-ω (10 ng/mL and 100 pg/mL) and one concentration for auto-Abs against IFN-β (10 ng/mL). Auto-Abs neutralizing lower and more physiological IFN concentration (100 pg/mL) were more frequently found than those neutralizing higher concentration (10 ng/mL). Indeed, 78.7% of patients (*n* = 74/96) presented neutralizing auto-Abs against IFN-α2 (100 pg/mL), 74% (*n* = 71/96) presented auto-Abs against IFN-ω (100 pg/mL), 50% (*n* = 48/96) had both neutralizing auto-Abs against IFN-α2 and IFN-ω (100 pg/mL) and only 12.5% of patients (*n* = 12/96) had neutralizing auto-Abs against IFN-β (10 ng/mL) (Figs. 1b and 2b). All auto-Abs were more frequently found in males, except anti-IFN-β auto-Abs, which were mostly found in women (Fig. 1a). The mortality rate at day 28 of patients who harboured auto-Abs neutralizing one or the other subtype of auto-Abs (Fig. 2a, b) or who carried one, two or three auto-Abs did not significantly differ (Fig. 2c).

### Women with auto-Abs neutralizing type I IFN

In the pioneer study of Bastard et al. [5], auto-Abs neutralizing type I IFN were detected in 94% of cases in men. Here, we found a higher proportion of women with positive auto-Abs, accounting for 21.9% (*n* = 21/96) of patients with neutralizing auto-Abs to type I IFN, consistent with other studies [8]. We thus further explored the characteristics of women with positive auto-Abs (Additional file 1: Table S1). As compared to auto-Ab negative women, women with neutralizing auto-Abs trended to be younger (45 (42–67) vs. 62 years (43–69), *p* = 0.090) and more frequently displayed an auto-immune background, with more frequent positive anti-nuclear antibody (ANA) (27.8 vs. 4.9%, *p* = 0.003). Three out of five patients with neutralizing auto-Abs to type I IFN and ANA displayed systemic lupus erythematosus serology with anti-DNA and/or anti-Sm Abs and one had anti-NOR90 Abs associated with scleroderma.

**Fig. 3:**
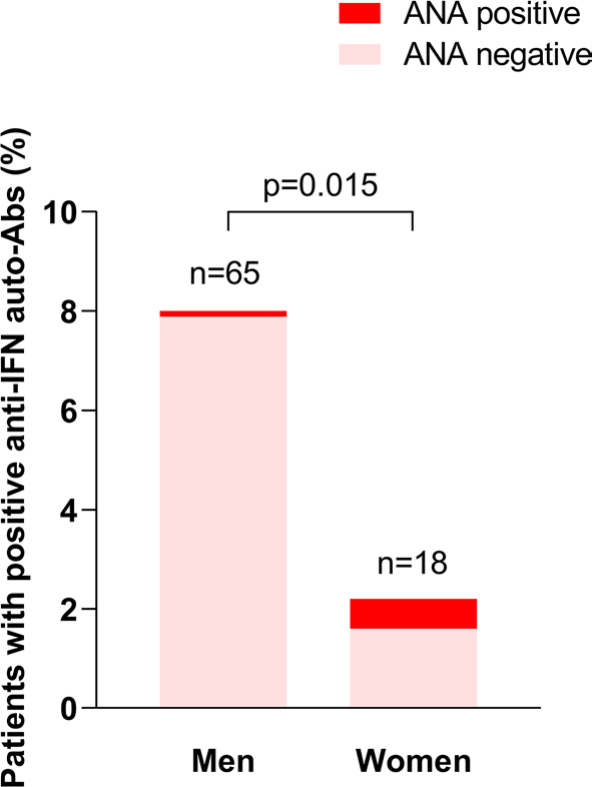
Positivity of anti-nuclear antibodies (ANA) according to gender in patients with positive anti-interferon (IFN) auto-antibodies (auto-Abs). ANA were screened in the serum of 812/925 patients of the cohort. ANA-positive patients are shown in red and ANA negative patients in pink. *P* value comes from the Fisher exact test

Interestingly, such difference regarding the distribution of ANA positivity according to anti-IFN auto-Abs status was not observed in men. Indeed, 2.6% (*n* = 13/504) of men with negative anti-IFN auto-Abs were tested positive for ANA, as compared with 1.6% of men with positive anti-IFN auto-Abs (*n* = 1/64; *p* > 0.99) (Fig. 3). Women with positive auto-Abs required more frequent invasive mechanical ventilation (71.4 vs. 46.8%, *p* = 0.040) and neuromuscular blockade (71.4 vs. 46.4%, *p* = 0.040) within 24 h of ICU admission, but mortality at day 28 and day 90 was not different.

Auto-Abs against IFN-α2 and IFN-ω were the most frequently found, but there was an unexpected higher rate of auto-Abs against IFN-β (33.3%).

## Discussion

We conducted a large prospective multicentre cohort study in which critically ill COVID-19 patients were screened for the presence of auto-antibodies neutralizing type I IFNs. The main results of our study are as follows: (1) auto-Abs were found in at least 10% of patients hospitalized in the ICU, consistent with previous studies; (2) critically ill patients who were found to be positive for auto-Abs against type I IFN did not have a different clinical presentation than others; and (3) they did not have a statistically different mortality at day 28 than patients without auto-Abs.

Our study included a large number of well-phenotyped critically ill COVID-19 patients and primarily aimed at studying the prognostic impact of auto-Abs neutralizing type I IFN. We found that 10% of patients had positive auto-Abs neutralizing type I IFN, consistent with several previous studies reporting positivity for neutralizing auto-Abs against type I IFNs, ranging from 3 to 19% in severe or critical cases [4, 5, 8–13, 15, 17, 19]. The number of auto-Ab-positive individuals might have been underestimated given the frequent use of corticosteroids that might lower the auto-Ab level or neutralization capacity. The majority of the patients of our study were positive for auto-Abs against IFN-α2 and IFN-ω, while patients with auto-Abs against IFN-β were less frequent. This result is consistent with previous studies showing that auto-Abs against IFN-β are scarce [11, 13]. Of note, in our study almost all positive patients against IFN-β were also positive for neutralizing auto-Abs against IFN-α2 and IFN-ω (*n* = 10/12, 83.3%). Such auto-Abs combination could be responsible for the lack of efficacy of sub-cutaneous IFN-β in severe COVID-19 [9].

We did not find any difference in terms of general characteristics and comorbidities between positive and negative patients. There was a trend for more positive patients being males but not to the extent to that reported in the study of Bastard et al. (94% positivity in males) [5].

Previous studies demonstrated that the detection of auto-Abs neutralizing type I IFNs in the overall population of COVID-19 patients is a risk factor for developing life-threatening COVID-19 pneumonia (i.e., requiring ICU admission) [5, 8, 9, 13]. We did not test the prevalence of auto-Abs positivity in healthy subjects of in mild or asymptomatic COVID-19 because of the inclusion criteria of our study, but Bastard et al. showed that anti-IFN auto-Abs are almost never found in these two populations with, respectively, 0.33% and 0% positivity rates [5]. Several studies demonstrated that auto-Abs pre-exist the viral infection [5, 12, 28], which we could not confirm as pre-infection samples were not available in our patients. Our study revealed that positive patients required higher FiO_2_ levels at ICU admission. However, PaO_2_/FiO_2_ ratios and need for ventilator support did not differ between groups, suggesting there was no major difference regarding the severity of respiratory disease between groups. Such findings are conflicting with those of other studies focusing on ICU patients reporting more frequent organ failures in patients with positive auto-Abs [12, 15].

Regarding the association between auto-Abs and mortality, there has been discrepancies in the published studies related to the population studied. In the overall COVID-19 population, the auto-Abs positivity is associated with an increased mortality in the majority of studies [9, 10]. However, when focusing on critically ill patients, small studies reported an association between auto-Abs positivity and mortality [9], while others failed to replicate such results [11, 12]. With a prospective and multicentre design and including a large number of critically ill COVID-19 patients, our study did not find a prognostic impact of auto-Abs positivity neither on 28-day (primary outcome measure), nor on 90-day mortality. Such findings in this cohort of critically ill patients do not obviate the key pathogenic role of auto-Abs against type I IFN in severe COVID-19, but suggest their presence might be more critical during the early phase of SARS-CoV-2 infection (i.e., before ICU admission) than when severe infection is constituted. Indeed, several factors may account for the lack of outcome difference between patients with and without positive auto-Abs. First, key clinical determinants, including organ failures, age, gender, associated comorbidities, have been demonstrated to have a major impact on the outcome of critically ill COVID-19 patients [27], and may have blunted the deleterious impact of auto-Abs neutralizing type I IFN in critical patients. Second, in our study, critically ill COVID-19 patients who were not found to be positive for auto-Abs against type I IFNs had the same clinical profile in terms of demographics, underlying comorbidities, and organ support than those who were positive, and eventually had the same outcomes. We therefore speculate that the similar clinical picture between patients with positive and negative auto-Abs might be explained by a common mechanism, i.e., an impaired type I IFN production or response. SARS-CoV-2 infection induces a strong innate immune response associated with the production of type I IFNs, triggered by the interaction of pathogen-associated molecular patterns and pattern recognition receptors [29]. However, compared to other severe viral infections (e.g., Influenza A H1N1 infections), severe COVID-19 was shown to be associated with a paradoxically lower type I IFN response. Indeed, severe COVID-19 patients have been characterized by type I IFN deficiency [30], associated with persistent viral load and a secondary exacerbated inflammatory response. The mechanisms underlying type I IFN deficiency in these critically ill patients who do not carry auto-Abs have to be investigated. Herein, we only studied the role of auto-Abs against type I IFN, but Zhang et al. [4] showed that 3.5% of critical COVID-19 patients carry significant inborn errors of type I IFN immunity related to loss of function variants, pointing out that a part of critical COVID-19 patients have an impaired IFN response unrelated to auto-Abs. A genetic assessment of all critically ill patients might lead to the identification of a higher number of patients with an identified type I IFN defect. SARS-CoV-2 variants have also been shown to sabotage the body’s IFN response through the production of immune-suppressive proteins [31]. An impaired type I IFN response thus seems to be a crucial determinant of severity at least in the early phase of SARS-CoV-2 infection, but maybe at a lesser extent in the latter stage when the innate immune response has been activated. Indeed, in vitro viral replication was inhibited when cells were pre-treated with type I IFN, but was not modified when exogenous IFN was added after cell infection [32]. This is consistent with the finding that treatment with IFN-β1a did not improve the course of the disease in hospitalized COVID-19 patients [33]. Such therapeutic strategies should thus rather be tested in the early stage of SARS-CoV-2 infection.

Twenty-two percent of women had positive auto-Abs, higher than the 6% rate reported in the first study of Bastard et al. [5]. Auto-Ab positive women displayed a peculiar phenotype, associating a younger age, more invasive mechanical ventilation requirement at ICU admission, and presented more frequently auto-Abs neutralizing IFN-β. Interestingly, they also had a more frequent auto-immune background. ANA have been reported in 14% of COVID-19 patients [14]. Auto-Abs against IFN have previously been identified in the pathogenesis of systemic lupus erythematous [34] but determining whether ANA positivity is triggered by SARS-CoV-2 infection or if patients with auto-immune background are more prompt to develop auto-Abs against type I IFNs will require more studies. Based on our results, we hypothesize that patients with auto-Abs may have two phenotypes, a first one represented by men over 60 years old without any auto-immune background, as described in the studies by the COVID human genetic effort consortium [5, 8], and another one, which is described here, represented by young women with auto-immune background. In both cases, in the ICU setting, auto-Abs against type I IFN are causal of life-threatening disease and seem to be associated with more severe disease at ICU admission in women, but not with mortality.

Our study has several strengths. Its multicentre and prospective design allowed for studying a representative sample of COVID-19 patients with little missing data. We could precisely assess the clinical phenotype and outcome of patients, who were followed up until day-90 of ICU admission. Finally, auto-Abs have been tested using the previously described reference method [5].

Our study also has limitations. Patients have been included from the beginning of the pandemics to May 2021. During this time-period, ICU admission strategies and patients’ management varied between the successive COVID-19 waves, introducing potential bias. The SARS-CoV-2 Alpha variant appeared in France and became predominant during the study inclusion period (January–May 2021) [35]. However, the distribution of positive patients did not change over time. We also did not record SARS-CoV-2 vaccination status as there was no anti-SARS-CoV-2 vaccine available at the time the study started. However, on May 1^st^ 2021, when the inclusion period ended, less than 10% of the French population had been fully vaccinated (https://covidtracker.fr/vaccintracker/), implying that the proportion of vaccinated patients in this cohort of critically ill patients was very low. The existence of a control group consisting in COVID-19 negative ARDS patients would have reinforced the findings of the study regarding the prevalence of auto-Abs. Performing immunological tests to assess the relationship between auto-Abs and host response to SARS-CoV-2 infection would have allowed to further explore the pathophysiological role of auto-Abs against type I IFNs in vivo in critically ill COVID-19 patients. Finally, auto-Abs were screened on only one blood sample during ICU stay, potentially underestimating the rate of auto-Abs positivity.

## Conclusions

In conclusion, auto-Abs against type I IFNs were found in at least 10% of critically ill COVID-19 patients, but in contrast with previous studies including both ICU and non-ICU patients, were not associated with increased mortality in this cohort of ICU patients. Further studies should aim at exploring an impaired type I IFN production or response in critically ill COVID-19 patients without positive auto-Abs, as they exhibit the same clinical features and outcomes than those who carry neutralizing auto-Abs. Routine screening of auto-Abs against type I IFN might be of interest before ICU admission to predict the risk of clinical worsening, as previously demonstrated, but seems to be of limited interest in the ICU setting to improve outcome prediction. Whether targeted treatment strategies should be initiated in patients with positive auto-Abs should be determined by future studies.

## Supplementary Information

**Additional file 1. Table S1.** Demographics and characteristics of women with severe SARS-CoV-2 infection (*n*=273) at intensive care unit admission, according to the presence of auto-antibodies against type I interferons.

## Data Availability

All data generated or analysed during this study are included in this published article and its supplementary files.

## Abbreviations

ANA: Anti-nuclear antibody
ARDS: Acute respiratory distress syndrome
Auto-Abs: Auto-antibodies
ICU: Intensive care unit
IFNs: Interferons
RT-PCR: Reverse transcriptase polymerase chain reaction
SAPSII: Simplified Acute Physiology Score
SOFA: Sepsis-related Organ Failure Assessment
TLR: Toll-like receptor
WHO: World Health Organization
