# Supplementary material for: Auto-antibodies against type I IFNs in > 10% of critically ill COVID-19 patients: a prospective multicentre study
Source: Ann Intensive Care. 2022 Dec 31;12:121. doi: 10.1186/s13613-022-01095-5 (PMC9803887; doi:10.1186/s13613-022-01095-5)
Supplement: Supplementary file 1 — Additional file 1. Table S1. Demographics and characteristics of women with severe SARS-CoV-2 infection (n=273) at intensive care unit admission, according to the presence of auto-antibodies against type I interferons. [file 13613_2022_1095_MOESM1_ESM.docx]

**Auto-antibodies against type I IFNs in >10% of critically ill COVID-19 patients**

Additional file 1

**Table S1**. Demographics and characteristics of women with severe SARS-CoV-2 infection (n=273) at intensive care unit admission, according to the presence of auto-antibodies against type I interferons

|  | **Negative anti-IFN auto-Abs (n=252)** | **Positive anti-IFN auto-Abs**  **(n=21)** | **p-value** |
| --- | --- | --- | --- |
| Age, year | 62 (53;69) | 45 (42;67) | 0.090 |
| BMI (kg/m²) | 32 (27;36) | 29 (26;33) | 0.430 |
| **Comorbidities** |  |  |  |
| Diabetes mellitus | 84 (33.3) | 4 (19) | 0.182 |
| Congestive heart failure^a^ | 13 (5.2) | 2 (9.5) | 0.327 |
| Vasculopathy | 21 (8.3) | 3 (14.3) | 0.412 |
| Hypertension | 133 (52.8) | 9 (42.9) | 0.381 |
| COPD | 11 (4.4) | 0 | 0.999 |
| Chronic kidney disease^b^ | 25 (9.9) | 2 (9.5) | 0.999 |
| ESRD requiring dialysis | 9 (3.6) | 2 (9.5) | 0.206 |
| Liver cirrhosis | 3 (1.2) | 0 | 0.999 |
| Current Smoking | 18 (7.1) | 1 (4.8) | 0.999 |
| Solid cancer | 8 (3.2) | 0 | 0.999 |
| Hematological malignancy | 4 (1.6) | 0 | 0.999 |
| Solid organ transplantation | 6 (2.4) | 1 (4.8) | 0.435 |
| HIV infection | 5 (2.0) | 0 | 0.999 |
| Long-term corticosteroids | 12 (4.8) | 2 (9.5) | 0.297 |
| Clinical frailty scale | 3 (2;4) | 3 (2 ;4) | 0.462 |
| **Treatment before ICU admission** | | | |
| Antibiotic | 125 (49.6) | 13 (61.9) | 0.279 |
| Corticosteroid therapy | 141 (56.0) | 12 (57.1) | 0.999 |
| NSAID | 5 (2.0) | 1 (4.8) | 0.387 |
| Tocilizumab | 12 (4.8) | 2 (9.5) | 0.297 |
| Convalescent plasma | 3 (1.2) | 0 | 0.999 |
| **Clinical characteristics at ICU admission** | | | |
| SAPS II | 33 (24;44) | 35 (28;54) | 0.304 |
| SOFA score | 4 (2;6) | 4 (3;6) | 0.148 |
| WHO CPS | 6 (6;8) | 8 (6;8) | 0.081 |
| Respiratory support |  |  |  |
| Oxygen therapy | 9 (3.6) | 1 (4.8) | 0.560 |
| High flow oxygen therapy | 153 (60.7) | 11 (52.4) | 0.472 |
| NIV | 81 (32.1) | 6 (28.6) | 0.718 |
| IMV | 118 (46.8) | 15 (71.4) | **0.030** |
| FiO_2_ (%) | 90 (60;100) | 100 (87;100) | 0.196 |
| PaO_2_ (mmHg) | 77 (60;94) | 72 (78.5;127.5) | 0.833 |
| PaCO_2_ (mmHg) | 26 (31;41) | 28 (26.5;35) | 0.299 |
| Arterial lactate (mmol/L) | 1 (1;2) | 1 (1;1.5) | 0.449 |
| PaO_2_/FiO_2_ ratio (mmHg) | 94 (70;141) | 89,5 (73;130) | 0.656 |
| ARDS | 231 (91.7) | 19 (90.5) | 0.693 |
| Shock | 51 (20.4) | 6 (28.6) | 0.404 |
| RRT | 25 (10) | 3 (14.3) | 0.464 |
| Bacterial co-infection | 32 (12.8) | 3 (14.3) | 0.741 |
| Antibiotherapy | 156 (61.9) | 17 (81) | 0.089 |
| Pulmonary embolism | 13 (5.2) | 0 | 0.608 |
| Neuromuscular blockade | 116 (46.4) | 15 (71.4) | **0.027** |
| Prone position | 122 (48.8) | 9 (42.9) | 0.601 |
| iNO | 9 (3.6) | 1 (4.8) | 0.560 |
| ECMO | 29 (11.6) | 4 (19.0) | 0.301 |
| **ICU management** |  |  |  |
| Invasive MV | 158 (62.7) | 14 (66.7) | 0.817 |
| MV duration, days | 16 (10;26) | 25 (13;31) | 0.200 |
| Live VFD at day 28 | 2 (0;5) | 3 (1;8) | 0.342 |
| Live VFD at day 90 | 2 (0;5) | 2 (0;5) | 0.829 |
| Shock | 131 (52.0) | 12 (57.1) | 0.82 |
| Duration of vasopressor support, days | 8 (4;14,5) | 10 (6;20) | 0.325 |
| Vasopressor-free days, 28 days | 12 (4;28) | 9 (5;15) | 0.495 |
| Vasopressor-free days, 90 days | 12 (4;28) | 9 (5;15) | 0.495 |
| RRT | 79 (31.3) | 8 (38.1) | 0.540 |
| Continuous sedation | 158 (62.7) | 15 (71.4) | 0.430 |
| Neuromuscular blockade | 155 (61.5) | 14 (66.7) | 0.646 |
| Prone position | 164 (65.1) | 14 (66.7) | 0.892 |
| iNO | 18 (7.1) | 1 (4.8) | 0.99 |
| ECMO | 43 (17.1) | 6 (28.6) | 0.234 |
| Duration of ECMO, days | 13 (6;32) | 14.5 (5;22) | 0.778 |
| Pulmonary embolism | 24 (9.5) | 2 (9.5) | 0.999 |
| Dexamethasone | 196 (77.8) | 15 (71.4) | 0.59 |
| Tocilizumab | 21 (8.4) | 2 (9.5) | 0,695 |
| Anti-IFNs auto-Abs type   - IFN-α2 10ng/mL - IFN-α2 100pg/mL - IFN-β 10ng/mL - IFN-ω 10ng/mL - IFN-ω 100pg/mL |  | 9 (42.9)  12 (57.1)  7 (33.3)  7 (33.3)  12 (57.1) |  |
| Anti-nuclear antibody | 11/225 (4.9) | 5/18 (27.8) | **0.003** |
| **Outcomes** |  |  |  |
| Mortality, 28-day | 49 (19.4) | 3 (14.3) | 0.774 |
| Mortality, 90-day | 59 (23.4) | 6 (28.6) | 0.600 |
| ICU mortality | 56 (22.2) | 6 (28.6) | 0.588 |
| Hospital mortality | 59 (23.4) | 6 (28.6) | 0.600 |
| Continuous values are shown as median (quartile 1-quartile 3); Qualitative values are shown as number (percentage); ^a^NYHA III-IV; ^b^GFR < 60mL/min/1.73m² ; **Bolded** values are significant at the <0.05 level.  *Definition of abbreviations :* BMI: body mass index ; COPD: chronic obstructive pulmonary disease ; ESRD: end-stage renal disease ;IMV: Invasive mechanical ventilation; NIV: Non-invasive ventilation; NSAID: non-steroidal anti-inflammatory drug; SAPSII: Simplified Acute Physiology Score II ; SOFA: Sequential Organ failure Assessment ; WHO CPS: World Health Organization clinical progression scale; ARDS: acute respiratory distress syndrome ; ECMO: extracorporeal membrane oxygenation; VFD: ventilator-free days; RRT: renal replacement therapy ; MV: mechanical ventilation; iNO: Inhaled Nitric Oxyde ; ECMO : extra-corporeal membrane oxygenation | | | |
